# Challenges Caused by Imported Cases Abroad for the Prevention and Control of COVID-19 in China

**DOI:** 10.3389/fmed.2021.573726

**Published:** 2021-05-20

**Authors:** Jianfei Zhu, Qingqing Zhang, Chenghui Jia, Shuonan Xu, Jie Lei, Jiakuan Chen, Yanmin Xia, Wenchen Wang, Xuejiao Wang, Miaomiao Wen, Hongtao Wang, Zhipei Zhang, Wuping Wang, Jinbo Zhao, Tao Jiang

**Affiliations:** ^1^Department of Thoracic Surgery, Tangdu Hospital, Air Force Military Medical University (Fourth Military Medical University), Xi'an, China; ^2^Department of Thoracic Surgery, Shaanxi Provincial People's Hospital, Xi'an, China; ^3^Department of Pulmonary and Critical Care Medicine, The First Affiliated Hospital of Xi'an Medical University, Xi'an, China; ^4^Department of Cardiothoracic Surgery, The First Affiliated Hospital of Xi'an Medical University, Xi'an, China

**Keywords:** COVID-19, global pandemic, imported case, epidemiological characteristics, prevention

## Abstract

**Background:** Overseas imported cases of COVID-19 continue to increase in China, so we conducted this study to review the epidemiological characteristics of these patients.

**Methods:** From February 26 to April 4, 2020, the imported cases from abroad were enrolled in this study. The effect of prevention countermeasures in curbing the spread of COVID-19 was assessed in this study. Moreover, we defined incubation period and confirmed time as from the date of leaving the epicenter to date of symptom onset and date of final diagnosed, respectively, and the interval of symptom onset to final diagnosed time was defined as diagnostic time. Categorical variables were summarized as numbers and percentages, and the difference among the variables were analyzed.

**Results:** For 670 cases imported from abroad, 555 were Chinese and 115 were foreigners. Apparently, confirmed cases had significantly decreased after China was compelled to temporarily suspend the entry of foreign passport holders with valid visas or residence permits; 6 days after implement of controlled measures, the daily new confirmed cases were reduced to 13 cases. Moreover, about 84.3% of patients (166/197) presented symptoms 1 week after leaving the epicenter, and notably seven patients (3.6%) had symptoms 2 weeks after leaving the epicenter. The median incubation period was 3.0 days (inter quartile range, 1.0 to 6.0), the 95th percentile was 11.6 days. Additionally, most of cases (92.9%) were detected positively of nucleic acid after symptom onset with 4 days, the median diagnostic time was 2.0 days (interquartile range, 1.0 to 3.0), and the 95th percentile of the distribution was 5.0 days. Finally, about 5.8% of patients were healthy carriers, and the median confirmed time of asymptomatic patients was 4.0 days (interquartile range, 2.0 to 9.0). The following variables might be associated with confirmed time: symptom type (*P* = 0.005), exported regions (*P* < 0.001), and symptom onset time (*P* < 0.001).

**Conclusions:** The prevention countermeasures for imported cases implemented by the Chinese government played an indispensable role in curbing the spread of COVID-19; the time of departure from epicenter could provide an estimate of the incubation period; and a confirmed time, 2-week quarantine period might need to be prolonged, while asymptomatic patients should be closely monitored.

## Introduction

The coronavirus disease (COVID-19) (1) epidemic broken out in Wuhan City of China in December 2019, and it had spread rapidly around the world (2) due to economic globalization. Internationally, on March 11, the epidemic spread worldwide in 114 countries or regions, a total of 118,319 confirmed cases and 4,292 deaths worldwide (3) were reported by WHO. Under this situation, WHO announced COVID-19 as a global pandemic (4). In China mainland, the epidemic was controlled effectively, on March 27, 20 provinces or autonomous regions had reported no domestic cases for more than 4 weeks (5), and some regions of China reduced the public health emergency to Level Three Response (6, 7).

To our disappointment, on February 26, the first imported cases were reported in Ningxia Hui Autonomous Region of China, imported from Iran (8). Epidemic prevention and control measures to guard against imported cases were brought out by the authorities, such as all entering people received temperature test and home quarantine. With the increasing number of imported patients confirmed, on March 18, professor Zhong, a top Chinese epidemiologist, suggested that all entry population from epicenter should undergo nucleic acid testing (9), which resulted in a continuous increase in the number of imported cases, as on March 26, the new imported cases reached 54 cases (10). To more powerfully curb the spread of COVID-19, on March 28, China was compelled to temporarily suspend the entry of foreign passport holders with valid visas or residence permits (11).

Therefore, prevention and treatment measures for imported cases based on the epidemiological characteristics of imported cases can be formulated. However, for imported cases, most of them could not provide the definite exposure time, which was indispensable for infected disease. In real life study, it was important to utilize the available information to predict the outcome of the imported cases. So, we conducted this study to analyze the epidemiological characteristics of COVID-19 cases imported from abroad by using the available information.

## Materials and Methods

### Study Design

**Figure 1:**
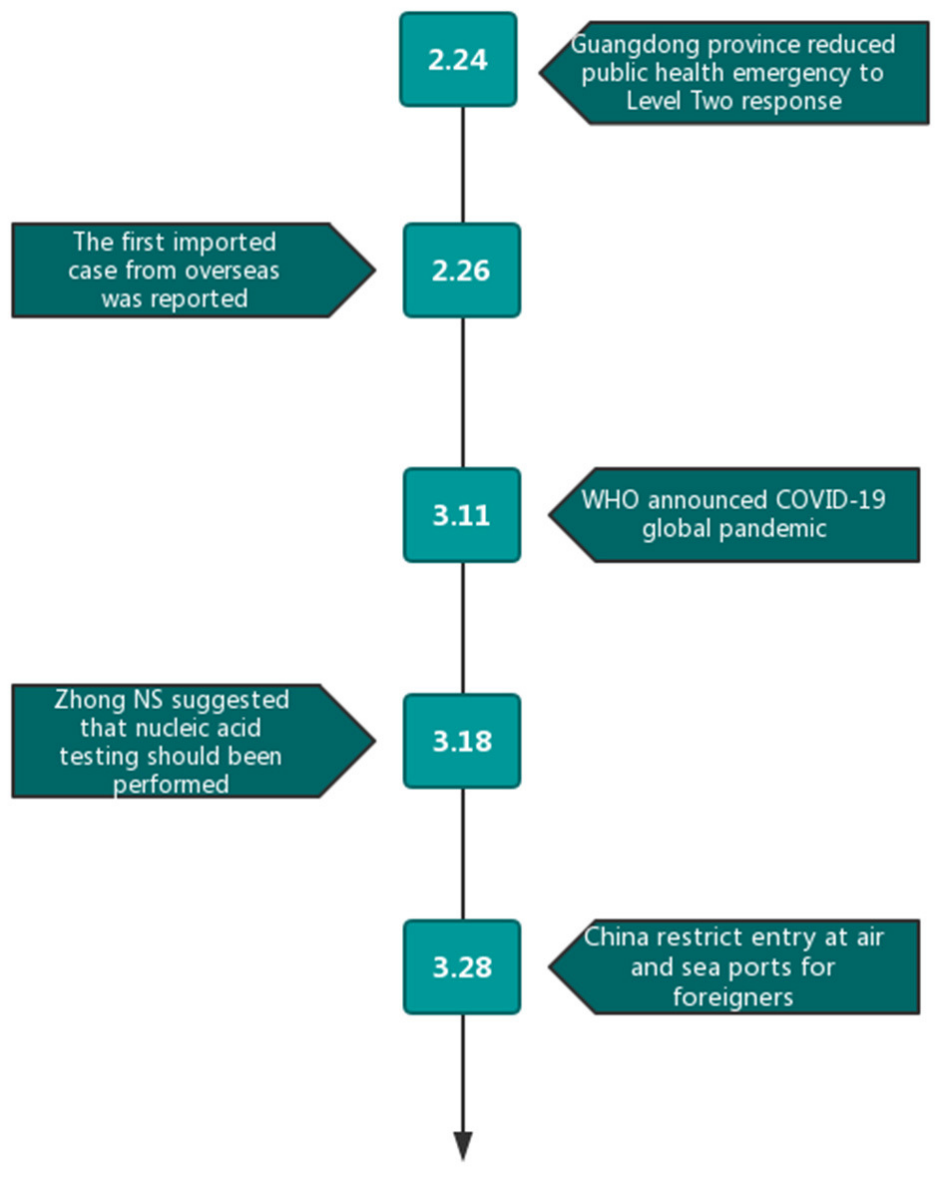
Date of occurrence of important events and implementation of preventive measures.

After strict prevention and treatment of COVID-19, on February 29, 20 provinces of China mainland announced no domestic COVID-19 cases (5). However, the confirmed cases outside of China continued to increase, and by March 11, WHO announced COVID-19 as a global pandemic (4). As a result, China's epidemic has reversed, changing from the exporting country to the importing one. The first imported case from Iran was reported on February 26, and China was compelled to temporarily suspend the entry of foreign passport holders with valid visas or residence permits on March 11 to curb the spread of COVID-19. The implementation date of critical public health prevention initiatives is displayed in Figure 1. This study was designed to review the epidemiological characteristics and outcomes of imported cases from abroad (from February 26 to April 4). The study was approved by the Review Board of the Air Force Medical University. Case enrollment needs to meet the following conditions: (1) imported from abroad; (2) underwent quarantine at designated places; (3) diagnosed by the results of nucleic acid test; and (4) adequate clinical information and available follow-up data.

### Data Collection

We collected the departure time and the country or region, the time of entry, symptoms, time of symptom onset, confirmed age and gender from the news reports and press releases reported by National Commission and Chinese Center for Diseases Control and Prevention. The degree of severity and diagnostic criteria refer to the 7th edition of the National New Coronavirus Pneumonia Diagnosis and Treatment Program. The data collection was performed by doctor Zhu, doctor Zhang, and doctor Jia, and any major disagreement among these three doctors was checked by the other two reviewers (doctor Xu and doctor Lei).

### Statistical Analysis

Due to lack of definite exposure time, we defined incubation period as from the date of leaving epicenter to date of symptom onset, and the interval of symptom onset to final diagnosed time was defined as diagnostic time (12). We also defined confirmed time as from date of leaving epicenter to date of final diagnosis. The incubation period and diagnostic time were estimated by fitting a Weibull distribution on the date of leaving epicenter and symptom onset. Categorical variables were summarized as numbers and percentages. *T* test were performed to compare the differences of incubation period among groups, and when the cases were not normal data distribution, Mann-Whitney U test or Kruskal–Wallis *H* test was used. It was considered statistically significant that *P* < 0.05. SPSS 20.0 was used to finish the above analyses.

## Results

### Clinical Characteristics

**Figure 2:**
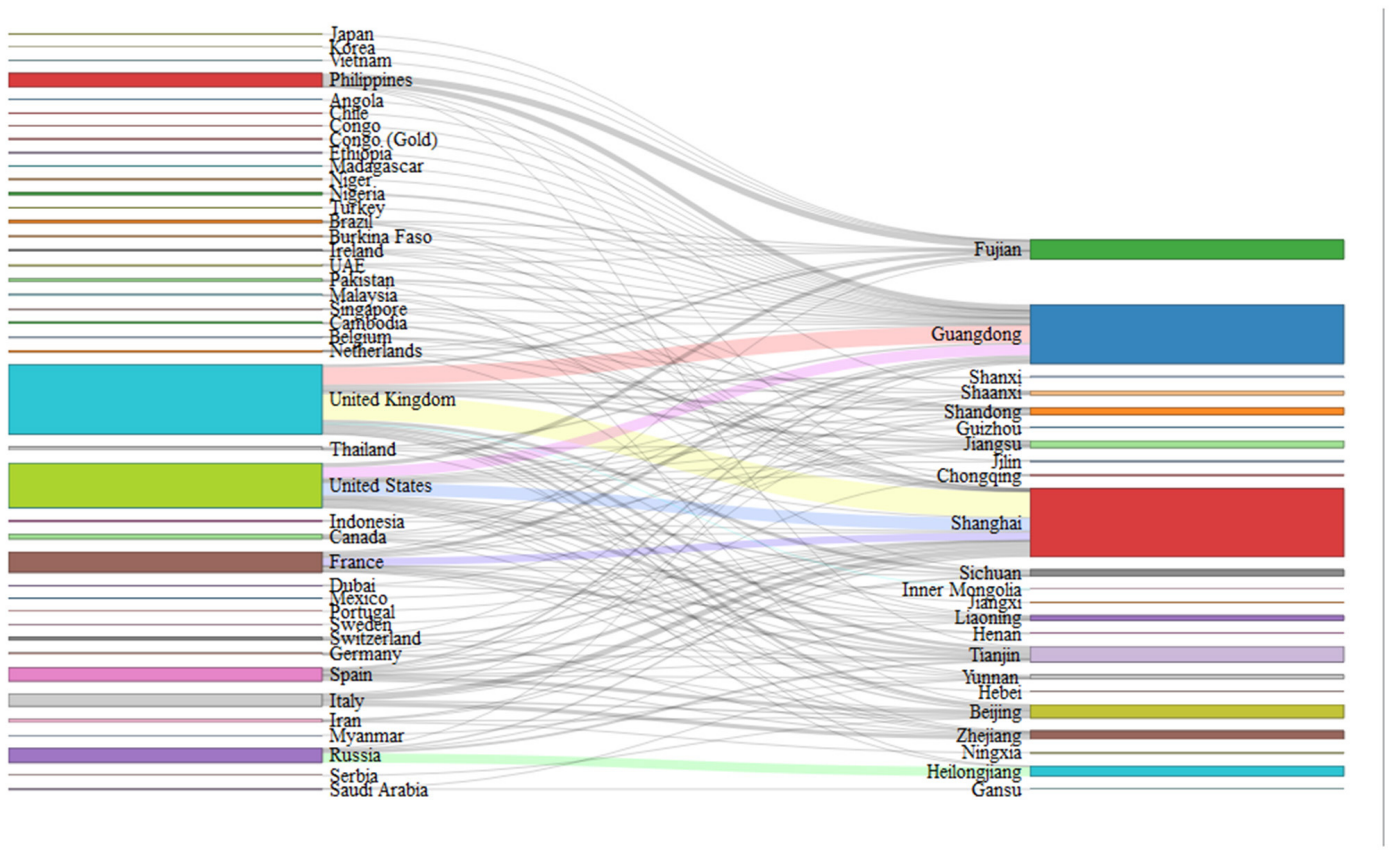
Distribution of exported countries and distribution of imported provinces.

**Figure 3:**
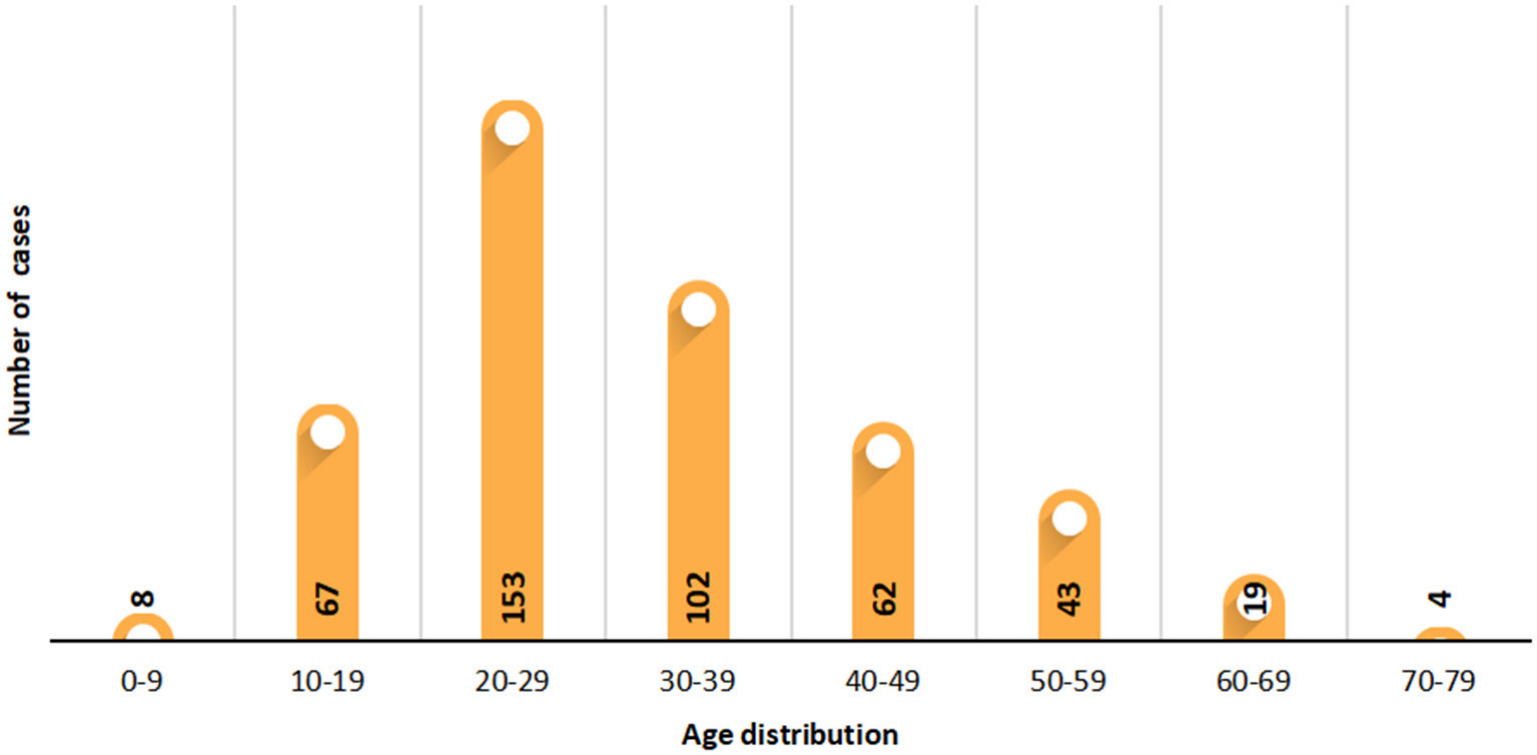
Age distribution of imported cases from abroad.

As of April 4, 2020, 1 week after China suspended the entry of foreign passport holders with valid visas or residence permits (February 28, 2020), cumulative imported cases from abroad increased to 913 cases, with 11 domestic cases related to imported cases. Finally, a total of 670 imported cases (Chinese: 555 cases and foreigners: 115 cases) were enrolled in this study. Europe was the main exported area (59.4%), followed by North America (20.9%) and South America (1.2%) (Table 1). The distribution of cases in exported countries and entry province, autonomous region or municipality was shown in Figure 2, and we show that United Kingdom and United States were main exported countries, and the main imported cities were metropolises, such as Shanghai and Guangzhou. For these cases, 269 cases were male, 209 were female, and 192 cases were unknown. Among 458 patients with clear age information, the oldest patient was 71 years old, the youngest was only 2 months old, and the median age of the patients was 30 years. Figure 3 showed that patients aged between 20 and 49 years accounted for 69.2% (317/458); the main reason may be that a large proportion of the overseas imported cases were students studying abroad (32.8%). The detailed information of the patients is listed in Table 1.

**Table 1:** Clinical characteristics of imported patients of COVID-19.

**Characteristics**	**Cases**	**Proportion**
Age, (median: 30 years, range from 2 month to 71 years)	*N* (670)	(%)
Gender	(null: 192)	28.7%
Male	269	40.2%
Female	209	31.2%
Age^a^	(null: 212)	31.6%
<30	228	34.0%
≥30	230	34.3%
**Time of entry**		
Before the global explosion	48	7.2%
Global explosion	7	1.0%
After the global explosion	615	91.8%
**Nationality**		
Foreigner	115	17.2%
Chinese	555	82.8%
**Region of cases exported**		
Asia	100	14.9%
Africa	24	3.6%
Europe	398	59.4%
South America	8	1.2%
North America	140	20.9%
**Occupation**		
International student	220	32.8%
Non-international students	450	67.2%
**Symptoms**		
Fever	107	16.0%
Respiratory symptoms	65	9.7%
Gastrointestinal symptoms	4	0.6%
Other symptom	26	3.9%
Without symptom	39	5.8%
Symptoms unknown	429	64.0%

a *Age: median age of all cases*.

### Public Health Interventions

**Figure 4:**
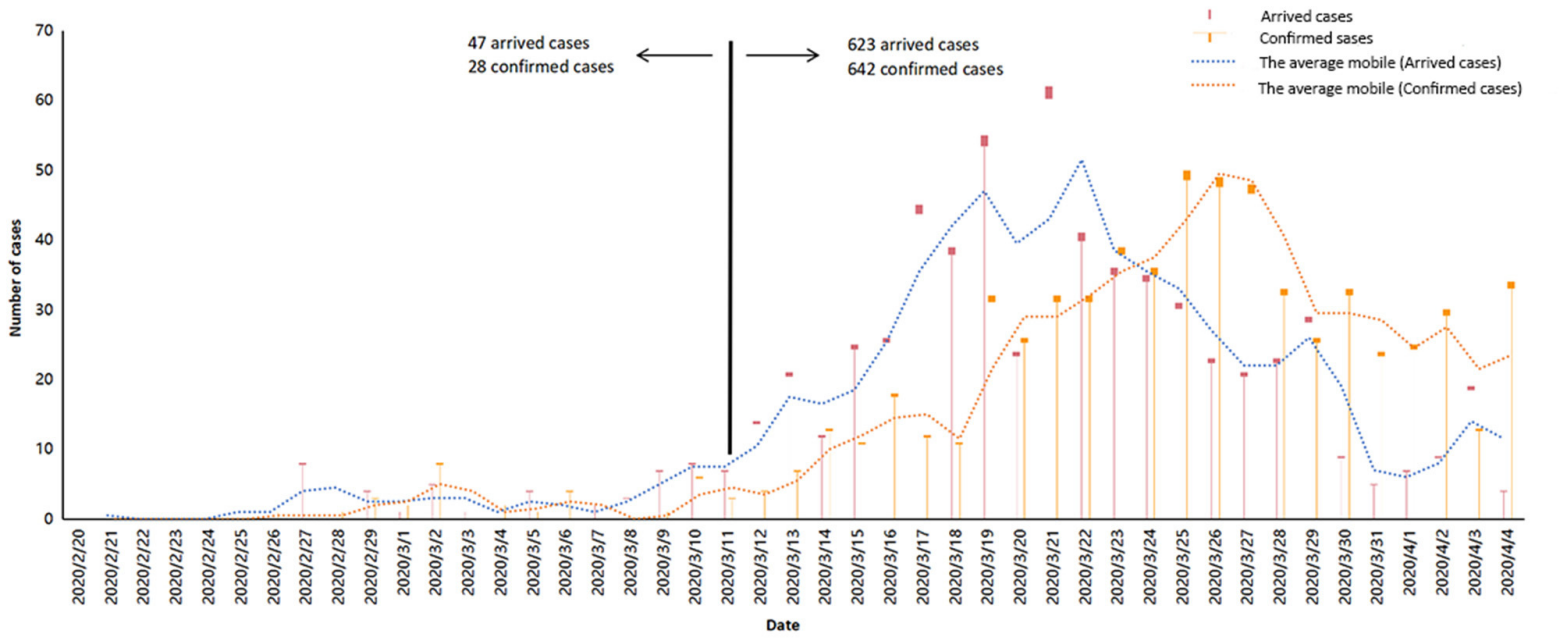
Time distribution of confirmed cases from abroad.

Since the first imported case from Iran was reported on February 26, China began to be concerned about the imported cases from abroad, with the reversal of domestic and foreign epidemics; on March 11, WHO announced COVID-19 as global pandemic. March 11 was an obvious watershed, the confirmed cases before and after were 28 cases and 642 cases, respectively. With the increase of confirmed cases, on March 18, Zhong Nanshan, a top Chinese epidemiologist, suggested that all entry population from epicenter should undergo nucleic acid testing, and 1 week after nucleic acid test was performed, the daily newly increased imported patients reached a peak with 50 cases. To more powerfully curb the spread of COVID-19, on March 28, China was compelled to temporarily suspend the entry of foreign passport holders with valid visas or residence permits. Apparently both arrived cases and confirmed cases had significant decreased, 6 days after implement of controlled measures, on April 4, and the daily new confirmed cases were reduced to 13 cases. The public health intervention events and related dynamic results are exhibited in Figure 4.

### Epidemiological Characteristics

**Figure 5:**
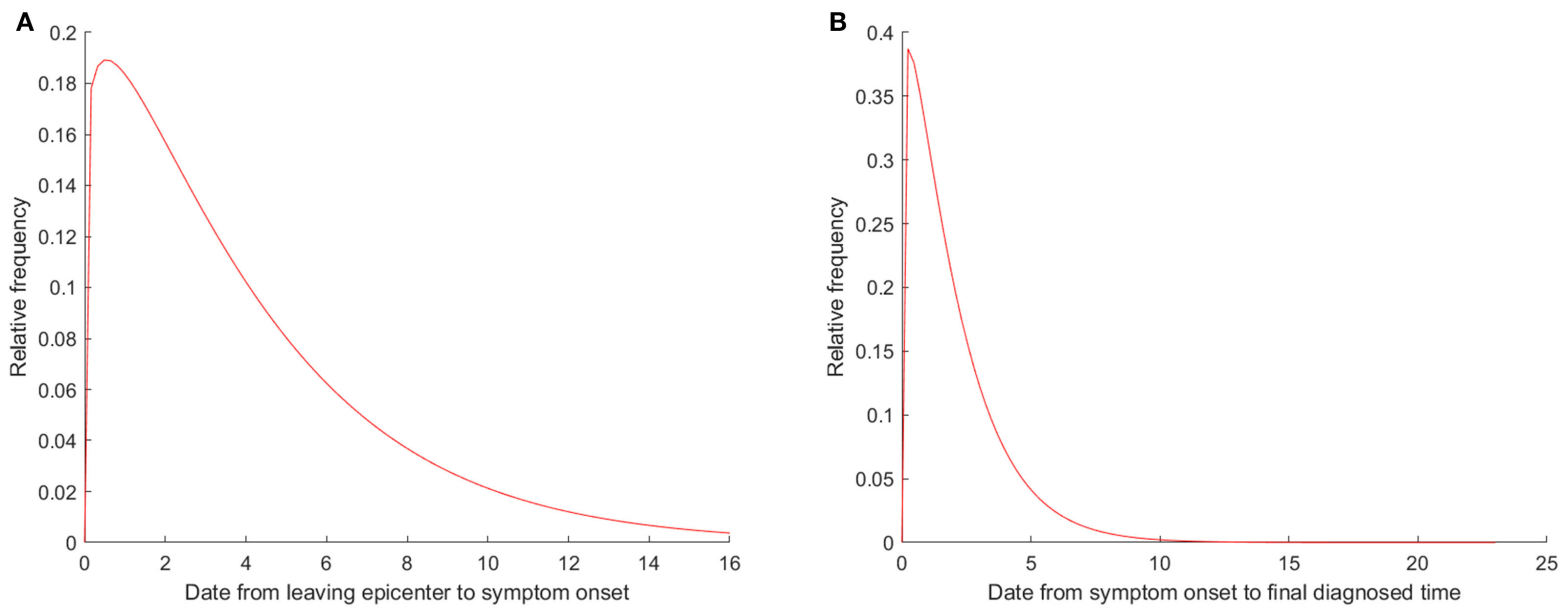
Estimation of incubation period and diagnostic time by fitting Weibull distribution. **(A)** Incubation period estimated on the date of leaving epicenter; **(B)** Diagnostic time were estimated on the date of symptom onset.

For symptomatic patients, 37 cases had symptoms before departure, 17 patients had symptoms on the day of departure, and 180 patients had symptoms after leaving the epicenter. About 84.3% of patients (166/197) presented symptoms 1 week after leaving the epicenter, and 7 patients (3.6%) had symptoms 2 weeks after leaving the epicenter. Interesting, the peak of symptom onset emerged was on the first day (42 patients). Incubation period was estimated by fitting Weibull distribution, and patients who presented symptoms before departure had been excluded from the analysis. The median incubation period was 3.0 days (interquartile range, 1.0 to 6.0), and the 95th percentile was 11.6 days (Figure 5A). Moreover, cases exported from Africa presented symptoms later than patients exported from other regions (*P* = 0.007), which may due to the outbreak time varied in different regions (Table 2). Studies related to the incubation period are listed in Table 3.

**Table 2:** Epidemiological characteristics of symptomatic patients imported from abroad.

**Variable**	**Incubation period (days)**	**IQI^b^**	***P***	**Diagnostic time (days)**	**IQI^b^**	***P***
In total (197)	3.0	(1.0, 6.0)		2.0	(1.0, 2.0)	
Gender			0.116			0.708
Male (87)	4.0	(2.0, 7.0)		1.0	(1.0, 2.0)	
Female (80)	3.0	(1.0, 5.0)		2.0	(1.0, 2.0)	
Age^a^			0.749			0.017
<27 (77)	4.0	(2.0, 6.0)		1.0	(0.0, 2.0)	
≥27 (84)	4.0	(2.0, 6.0)		2.0	(1.0, 2.0)	
Occupation			0.570			0.044
International student (71)	4.0	(1.0, 6.0)		1.0	(1.0, 2.0)	
Non-international students (126)	3.0	(1.0, 6.0)		2.0	(1.0, 2.25)	
Symptoms			0.067			0.307
Fever (89)	4.0	(2.0, 6.0)		2.0	(1.0, 2.0)	
Respiratory symptoms (41)	2.0	(1.0, 6.0)		1.0	(1.0, 2.0)	
Gastrointestinal symptoms (3)	3.0	(2.5, 3.0)		1.0	(1.0, 1.5)	
Other symptom (17)	4.0	(1.0, 7.0)		1.0	(0.5, 2.0)	
Symptoms unknown (47)	1.0	(1.0, 5.0)		2.0	(1.0, 2.0)	
Nationality			0.805			0.815
Foreigner (27)	2.0	(1.0, 8.0)		1.0	(1.0, 2.0)	
Chinese (170)	3.0	(1.0, 6.0)		2.0	(1.0, 2.0)	
Region of cases exported			0.007			0.309
Asia (33)	2.0	(0.0,5.0)		2.0	(1.0, 3.0)	
Africa (7)	8.0	(3.0,14.0)		1.0	(1.0, 2.0)	
Europe (114)	3.0	(1.0,5.0)		1.5	(1.0, 2.0)	
South America (3)	5.0	(5.0,5.5)		1.0	(1.0, 2.0)	
North America (40)	4.0	(2.0,6.0)		2.0	(1.0, 3.0)	

a *Age, median age of symptomatic cases.*

b *IQI, interquartile intervals*.

**Table 3:** Study list of incubation period of COVID-19 estimation.

**References**	***N***	**Distribution**	**Mean, days (95% CI)**	**Median, days (95% CI)**	**Percentiles**
Zhang et al. (13)	49	Log-normal	5.2 (1.8, 12.4)	-	95th: 10.5
Li et al. (14)	Null	Gamma	7.2 (6.8, 7.6)	-	-
Leung et al. (15)	175	Weibull	(Travelers to Hubei) 1.8 (1.0, 2.7) (Non-travelers) 7.2 (6.1, 8.4)	-	(Travelers to Hubei) 95th: 3.2 (Non-travelers) 95th: 14.6
Qian et al. (16)	262	-	6.7	-	-
Ping et al. (17)	758(93)	Log-normal	-	8.06 (6.9, 9.4).	95th: 21.9
Qian et al. (16)	91	-	-	6 (3.0, 8.0)	-
Wang et al. (18)	483	Logarithm normal	7.4	7.0	-
Lu et al. (19)	265	Weibull	6.4 (5.3, 7.6)	-	5th: 1.0 95th: 13.1
Ki et al. (20)	28	-	3.9 (0, 15.0)	3.0	-
Guan et al. (21)	1,099	-	-	4.0 (2.0, 7.0)	-
Li et al. (14)	10	Log-normal	5.2 (4.1, 7.0)	-	95th:12.5(9.2, 18)
Backer et al. (22)	88	Weibull Gamma Lognormal	6.4 (5.6, 7.7) 6.5 (5.6, 7.9) 6.8 (5.7, 8.8)	-	95th:10.3 (8.6, 14.1) 95th:11.3 (9.1, 15.7) 95th:13.3 (9.9, 20.5)
Lauer et al. (23)	181	Outside mainland China (*n* = 108).	-	5.5 (4.4, 7.0)	95th: (2.1, 14.7)
Lauer et al. (23)	181	Inside mainland China (*n* = 73)	-	4.8 (4.2, 5.6)	95th: (2.5, 9.2)
Lauer et al. (24)	181	Lognormal	-	5.1 (4.5,5.8)	97.5th: 11.5 (8.2,15.6)
Linton et al. (25)	Excluding Wuhan residents (*n* = 52)	Lognormal Weibull Gamma	5.0 (4.2, 6.0) 5.4 (4.3, 6.6) 5.3(4.3, 6.6)	4.3(3.5, 5.1) 4.7(3.6, 5.8) 4.7(3.8,5.7)	95th:10.6 (8.5, 14.1) 95th:12.0 (9.8, 15.6) 95th:11.3 (9.2, 14.5)
Linton et al. (25)	Including Wuhan residents (*n* = 158).	Lognormal Weibull Gamma	5.6 (5.0, 6.3) 5.8 (5.2, 6.5) 6.0 (5.3, 6.7)	5.0(4.4, 5.6) 5.3(4.7, 6.0) 5.6(4.9, 6.4)	95th:10.8 (9.3, 12.9) 95th:11.0 (9.6, 12.9) 95th:11.7 (10.3, 13.4)
Jing et al. (12)	1,211	Weibull	8.6 (8.0, 9.23)	8.1 (7.4, 8.9)	90th:14.7 (14.0, 15.3) 99th:20.6(19.5, 21.6)
Han et al. (24)	59	Monte Carlo simulation	5.8	5.0	-

Furthermore, we also researched the serial interval of symptom onset to final diagnosed (positive results of nucleic acid test). Two patients presented symptoms after final diagnosed; most cases (92.9%) were detected positively of nucleic acid after symptom onset with 4 days. For symptomatic patients, the median diagnostic time was 2.0 days (interquartile range, 1.0 to 3.0), the 95th percentile of the distribution was 5.0 days, it was showed in Figure 5B, furthermore, age and occupation were associated with diagnostic time (*P* = 0.017 and *P* = 0.044) (Table 2).

**Figure 6:**
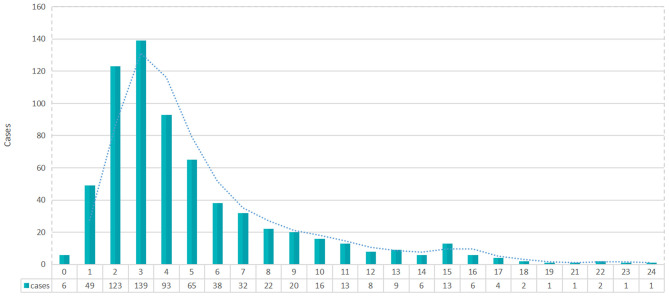
Distribution of confirmed time after confirmed cases leave the epidemic area.

Finally, the median confirmed time of all patients was 4.0 days (interquartile range, 2.0 to 6.0). It was worth noting that about 5.8% patients were healthy carriers, and the median confirmed time of asymptomatic patients was 4.0 days (interquartile range, 2.0 to 9.0). Figure 6 showed that the peak of confirmed time was on the third day (139 cases); there were still 37 patients (5.5%) who were final diagnosed 2 weeks after leaving the epicenter. The following variables might be associated with confirmed time: symptom type (*P* = 0.005), exported regions (*P* < 0.001), and symptom onset time (*P* < 0.001) (Table 4).

**Table 4:** Confirmed time of imported COVID-19 patients.

**Variable**	**Confirmed time (median, days)**	**IQI^b^**	***P***
In total (670)	4.0	(2.0, 6.0)	
Gender			0.776
Male (269)	4.0	(2.0, 7.0)	
Female (209)	4.0	(2.0, 7.0)	
Age^a^			0.281
<30 (228)	3.0	(2.0, 6.0)	
≥30 (230)	4.0	(2.0, 7.0)	
Occupation			0.1
International student (220)	4.0	(3.0, 6.0)	
Non-international students (450)	4.0	(2.0, 6.25)	
Symptoms			0.005
Fever (107)	5.0	(3.0, 8.0)	
Respiratory symptoms (65)	3.0	(2.0, 6.0)	
Gastrointestinal symptoms (4)	4.0	(3.25, 4.75)	
Other symptom (26)	3.0	(2.0, 5.5)	
Without symptom (39)	4.0	(2.0, 9.0)	
Symptoms unknown (429)	4.0	(3.0, 6.0)	
Nationality			0.066
Foreigner (115)	4.0	(3.0, 8.0)	
Chinese (555)	4.0	(2.0, 6.0)	
Region of cases exported			<0.001
Asia (100)	4.0	(2.0, 7.0)	
Africa (24)	8.0	(4.25, 13.75)	
Europe (398)	3.0	(2.0, 5.0)	
South America (8)	8.5	(7.25, 9.75)	
North America (140)	4.0	(3.0, 7.0)	
Symptoms onset			<0.001
Symptom onset is clear (234)	4.0	(3.0, 7.0)	
Symptoms during isolation (79)	6.0	(4.0, 8.0)	
Asymptomatic (39)	4.0	(2.0, 9.0)	
Unknown time of symptoms (318)	3.0	(2.0, 5.0)	

a *Age, median age of all cases.*

b *IQI, interquartile intervals*.

## Discussion

With the global outbreak of COVID-19, the confirmed cases and deaths continue to rise globally (26–29). In China, the domestic epidemic was controlled effectively but the challenge of cross-border COVID-19 transmissions emerged. To our knowledge, this study is the first designed to review the epidemiological characteristics of COVID-19 cases imported from abroad in China. Most of them were exported from Europe and North American, which might be related with the economic and academic exchanges between China and these regions.

Internationally, according to statistics, about 145 million tourists traveled to China from abroad in 2019 (30), and most of them came from Europe and North America. We also know that Europe and North America were the most popular areas for Chinese students (31). The distribution of exported regions of COVID-19 was associated with above data, and in this study, about 74.3% cases came from Europe and North America and nearly one-third of exported cases were Chinese overseas students. As exported country, previous studies have shown most cases from China had spread to Asian neighbors, followed by Europe (32). In our study, although there were few cases (4.8%) exported from Africa and South America, the imported cases in these two regions could not be ignored. On February 26, the first imported case from Iran was reported; after that, the confirmed increased steadily, as on March 11, COVID-19 was defined as a global pandemic, and the imported cases increased sharply.

Incubation period, from the earliest exposure time to the time of symptoms onset, was necessary for preventing and controlling the epidemic, so numerous studies were focused on this issue. The median incubation period was ranged from 3.9 days to 8.6 days due to the different definition of exposure time (13, 14, 16–19, 21–25, 33, 34). However, in a real world study, most patients could not provide the specific exposure time. It was significant to estimate the incubation period by using the available information. Jing et al. (12) estimated the median incubation period of COVID-19 was 8.13 days (95% CI: 7.37–8.91), and they defined it as from the time of departure from Wuhan City to the symptom onset by using the well-known renewal theory in probability. For the imported cases, preventive measures were formulated based on the incubation period; unfortunately, we could not obtain the definite exposure time, and we supposed that the time of leaving epicenter could predict the progression of COVID-19. In this study, we found that the median incubation of the imported COVID-19 cases was 3.0 days (interquartile range, 1.0 to 6.0) after they leaving the epicenter, shorter than the previous study. The following reasons can explain its short incubation period: a minority of them were infected on the way to China, or the mutations in the novel coronavirus (35). Notably, there were still 3.6% of patients who had symptom 2 weeks after leaving the epicenter, which indicated that the 2-week quarantine period might need to be prolonged. The outbreak of sporadic epidemics since mid-2020 were related to the onset of imported cases after the 2-week quarantine period; therefore, the Chinese government had formulated a 2-week plus 7-day quarantine policy for overseas cases.

We also explored the optimal nucleic acid detection time for imported cases in this study. For the symptomatic patients, the median diagnostic time was 2.0 days after they presented symptoms. A study from South Korea (20) showed that the median of symptom-onset to diagnosis was 5.2 days, the longest time of symptom-onset to diagnosis was 16.0 days reported by Xiao, and a total of 301 patients were analyzed (36). In this study, the positive results of nucleic acid test were detected earlier than the related studies, which might be due to that all inbound population from epicenter were suggested to underwent nucleic acid test when they arrived in China.

Moreover, our experience of fighting the COVID-19 indicated that public health intervention could reduce its transmission (37). The public health intervention, including strictly enforced segregation and travel bans, entry screening, suspension of public transportation in the city, closure of entertainment venues, and bans on public gatherings, played an important role in controlling the epidemic (38–40). One study proved that the Wuhan City shutdown delayed the spread of COVID-19 to other cities for 2.91days (95% CI: 2.54–3.29) (39). Therefore, on March 28 (11), China was compelled to temporarily suspend the entry of foreign passport holders with valid visas or residence permits. Apparently both arrived cases and confirmed cases had significantly decreased.

Finally, with asymptomatic patients, because the condition was hidden and might be contagious, we should be more vigilant. In our study, 39 asymptomatic patients were detected during their quarantine. A study about a health carrier from Wuhan caused widespread concern, she transmitted new coronavirus to her five family relatives, but she did not present any symptoms during her disease course (41). A series of subsequent studies confirmed that asymptomatic patients were infectious (42–45). The median of confirmed of asymptomatic patients was 4.0 days, basically consistent with symptomatic patients. Therefore, nucleic acid testing of all immigrants was essential for screening asymptomatic patients.

The shortcoming of this study was that the retrospective study did not include all people returning from overseas (243 cases were lost), so it could not represent the epidemiological characteristics of all imported confirmed cases; secondly, we estimated the incubation period for COVID-19; the departure time from the epidemic area was recognized as the exposure time, which may lead to a reduction in the real incubation period. Third, we could not quantify that interventions had multiple indirect effects on changes in human behavior. Finally, most patients were still in hospital, so the clinical outcomes had not been analyzed.

It is important to plan for the early stages of imported cases from overseas during a pandemic to avoid a national outbreak. In this study, we investigated the epidemiological characteristics of COVID-19 imported from overseas and observed interventions implemented by the government. We found that the departure time from epicenter could be used to predict the progression of the COVID-19. Promulgation of a ban on entry, a combination of quarantine, and quarantine methods was effective. For imported cases, the 2-week quarantine period might need to be prolonged and asymptomatic patients should be closely monitored.

## Data Availability Statement

The original contributions presented in the study are included in the article/supplementary material, further inquiries can be directed to the corresponding author/s.

## Ethics Statement

The studies involving human participants were reviewed and approved by Ethics Committee of Tangdu Hospital. The patients/participants provided their written informed consent to participate in this study.

## Author Contributions

TJ, JZha, and WuW participated in study design and study conception. JZhu, QZ, CJ, SX, JL, HW, and ZZ performed data analysis. JZhu, QZ, CJ, SX, JC, YX, WeW, XW, and MW recruited patients. JZhu, QZ, CJ, WuW, and JC drafted the manuscript. All authors provided critical review of the manuscript and approved the final draft for publication.

## Conflict of Interest

The authors declare that the research was conducted in the absence of any commercial or financial relationships that could be construed as a potential conflict of interest.
